# Automated Classification of Severity in Cardiac Dyssynchrony Merging Clinical Data and Mechanical Descriptors

**DOI:** 10.1155/2017/3087407

**Published:** 2017-02-19

**Authors:** Alejandro Santos-Díaz, Raquel Valdés-Cristerna, Enrique Vallejo, Salvador Hernández, Luis Jiménez-Ángeles

**Affiliations:** ^1^Bioengineering Department, Instituto Tecnológico y de Estudios Superiores de Monterrey, Campus Ciudad de México, Mexico City, Mexico; ^2^Neuroimaging Laboratory, Electrical Engineering Department, Universidad Autónoma Metropolitana Iztapalapa, Mexico City, Mexico; ^3^Centro Medico ABC (American British Cowdray Hospital), Mexico City, Mexico; ^4^Nuclear Cardiology Department, Instituto Nacional de Cardiología “Ignacio Chávez”, Mexico City, Mexico; ^5^Engineering in Biomedical Systems Department, Faculty of Engineering, Universidad Nacional Autónoma de México, Mexico City, Mexico

## Abstract

Cardiac resynchronization therapy (CRT) improves functional classification among patients with left ventricle malfunction and ventricular electric conduction disorders. However, a high percentage of subjects under CRT (20%–30%) do not show any improvement. Nonetheless the presence of mechanical contraction dyssynchrony in ventricles has been proposed as an indicator of CRT response. This work proposes an automated classification model of severity in ventricular contraction dyssynchrony. The model includes clinical data such as left ventricular ejection fraction (LVEF), QRS and P-R intervals, and the 3 most significant factors extracted from the factor analysis of dynamic structures applied to a set of equilibrium radionuclide angiography images representing the mechanical behavior of cardiac contraction. A control group of 33 normal volunteers (28 ± 5 years, LVEF of 59.7% ± 5.8%) and a HF group of 42 subjects (53.12 ± 15.05 years, LVEF < 35%) were studied. The proposed classifiers had hit rates of 90%, 50%, and 80% to distinguish between absent, mild, and moderate-severe interventricular dyssynchrony, respectively. For intraventricular dyssynchrony, hit rates of 100%, 50%, and 90% were observed distinguishing between absent, mild, and moderate-severe, respectively. These results seem promising in using this automated method for clinical follow-up of patients undergoing CRT.

## 1. Introduction

Heart failure (HF) is a cardiovascular disease with one of the highest morbidity, mortality, and hospital admissions worldwide among those over 55 years of age [1–5]. According to the American Heart Association, between 2009 and 2012 more than 5.7 million of patients, over the age of 20, suffered from HF in the United States. Also, more than 915,000 new cases are diagnosed annually with a mortality rate of 29.6% after 1 year of diagnosis and 52.6% after 5 years of diagnosis [6]. In a Sweden report, the case-fatality rate of HF within 5 years was of 59%, similar to 58% of the patients with the most common types of cancer (lung, colorectal, prostate, and bladder) [7]. However, HF affects not only individuals but also government expenses accounting for 1-2% of the global health budget [8, 9]. These mortality and financial statistics highlight the public health care burden of HF.

HF is a syndrome affecting the performance of the heart as a pump, which at the beginning reduces the capability for exercising and progressively may develop into conditions such as pulmonary and systemic congestion. It creates a progressive deterioration in the structure and function of the heart as well as development of arrhythmias, leading to the first cause of morbidity and mortality among the disease [10, 11]. It has been shown that 30% of people with severe HF show electric conduction disorders and develop ventricular contraction dyssynchrony, with an increase in left ventricle malfunction [12].

Today, HF is quantified into four levels according to the patient's functional classification where physical capability is evaluated [13]. Functional class has an important prognostic value and it is used as decision criteria for therapeutic intervention, being pharmacological, surgical, or both. Periodic evaluation of functional class allows following the evolution and response to treatment [14].

Cardiac Resynchronization Therapy (CRT) is an accepted treatment for patients with HF, impaired left ventricular function, and wide QRS complex. It is based on implantable devices that send electrical impulses to the lower chambers of the heart and help them beat together synchronously [14]. Guidelines from the American College of Cardiology/American Heart Association/Heart Rhythm Society recommend CRT for HF patients with a functional class III or IV, left ventricle ejection fraction (LVEF) < 35%, and an electrocardiogram trace with a QRS complex greater than 120 milliseconds [14, 15]. Efficacy of CRT has been demonstrated in multiple trials, showing a significant improvement in 6-minute walk distance, quality of life, peak oxygen uptake, functional classification, hospital admissions, and mortality among patients with left ventricle (LV) malfunction, and ventricular electric conduction disorders [16, 17]. The improvement in LVEF seems to correlate with a better long-term survival [18]. However, in HF patients under CRT, reverse remodeling is able to predict long-term outcome with higher reproducibility and predictive power than changes in LVEF. Thus, reverse modeling defined as the changes in LV end-systolic volume relative to baseline (≥15%) is currently considered the strongest predictor of mortality and HF-hospitalization [19].

In spite of this, reports on the benefits of CRT show that 30–40% of the patients fail to respond to the therapy [15], when applying the conventional criteria. It was suggested that the extent of the viable or infracted myocardium, the LV lead placement, and the presence of mechanical contraction dyssynchrony in ventricles could be related to the success of CRT [20, 21].

Different modalities of medical imaging based on the analysis of mechanical contraction of ventricles have proposed indicators to quantify ventricular contraction dyssynchrony, for example, Doppler Tissue Imaging (DTI) that has been used extensively to evaluate left ventricle dyssynchrony and predict its response [22]. Simple DTI uses the basal segments of a four-chamber view from the apical third to measure the delay between septal and lateral walls contraction. It was hypothesized that a delay higher than 65 milliseconds predicts a favorable response to CRT and to the final systolic volume of left ventricle. However, the multicenter study, PROSPECT Trial, showed that the dyssynchrony measure, based on DTI, did not achieve the right sensitivity and specificity to identify CRT responders [23].

With the use of Cardiac Magnetic Resonance (CMR) several methods have been proposed to evaluate left ventricle dyssynchrony [24]. Some of them use cine myocardial tagging and strain imaging, a technique that presents a tridimensional map that changes in color reflecting the timing and distribution of circumferential strains during mechanical contraction and relaxation. In a patient with ventricular contraction abnormality, the map will show heterogeneity in color coding mainly related to the septum and the lateral wall [16]. Nevertheless, the time processing to achieve this map is too high and it might suffer from meaningful degradation. Furthermore, CMR evaluations can be performed safely only in patients with particular pacemakers and implantable cardioverter-defibrillator systems by using a protocol based on device selection, appropriate device reprogramming, and close monitoring during the scan. Thus, MRI should only be performed in patients with adequate subspecialist supervision and monitoring staff, where the potential benefit clearly outweighs the risks [25].

Gated SPECT myocardial perfusion is a radionuclide-based imaging method that allows assessment of left ventricular (LV) mechanical dyssynchrony using Fourier phase analysis [26, 27]. This technique fits the temporal evolution of the LV to the first harmonic of the Fourier Transform and extracts the phase angle that represents the time of the onset of mechanical contraction across the LV. The standard deviation and width (encompassing 95% of the samples) of the phase histogram have been validated as indices of LV dyssynchrony showing a moderate correlation with electrical dyssynchrony and preliminary results suggest that they can be used in predicting CRT response [28, 29].

Equilibrium Radionuclide Angiography (ERNA) is another nuclear medicine option to evaluate ventricular function and dyssynchrony. It represents the spatial distribution of a radiotracer and relates pixel intensity to ventricular volume. Fourier phase analysis applied to ERNA images consists of adjusting each pixel's intensity temporal evolution (time-activity curve or TAC) to the first harmonic component of the Fourier Transform (FT). Then, phase angles representative of the TAC behavior is extracted from these components, and a map (phase image) representing the ventricular contraction sequence is constructed [30]. Several indices, taken from the statistical distribution of the phase angles, have been proposed to detect abnormal contraction patterns [31].

ERNA allows serial evaluations for both ventricles function with high accuracy and temporal resolution [30]. Fourier phase analysis applied to ERNA has been used to evaluate interventricular and intraventricular dyssynchrony with high accuracy and reproducibility [32, 33]. An early study showed the prognostic value of intraventricular dyssynchrony as an independent predictor of a cardiac event in idiopathic dilated cardiomyopathy patients [34] and recently has been described as highly predictive for acute volumetric response to CRT in HF patients [35, 36]. However, Fourier phase analysis of ERNA images using only one Fourier Transform harmonic has its limitations, since it assumes periodic TACs and a smooth transition between the first and last frame of the dynamic images series. These drawbacks are more prominent in the regions with severe contraction pattern abnormalities.

Factor Analysis of Dynamic Structures (FADS) have also been suggested as a valuable tool to detect abnormalities in ventricular contraction [37, 38]. It is applied to ERNA images to extract those TACs associated with the physiological behavior of a particular region and assumes that there are pixel clusters with the same temporal evolution, which define their morphology. Therefore, FADS determine the TACs (coefficients) of pixel groups with the same behavior, in addition to their geometry and spatial location (factors) [39]. In previous works carried out by our group, we analyzed the contribution and spatial distribution of the most significant factors of a dynamic series of ERNA images. We introduced an alternative method to reconstruct a map for the sequence of ventricular contraction. In [40] it was noted that more than 90% of the information contained in an image series was represented by the three most significant factors (3-MSF) and that the eigenvalue of the third factor increases significantly whenever an abnormality of the contraction pattern was analyzed. Also, a detailed analysis of the scatter plots of the 3-MSF displayed the importance of the third factor to adequately separate regions having an abnormal contraction pattern. In [41] we proposed a normality index of ventricular contraction based on the likelihood between the probability density function model of the 3-MSF extracted from FADS of a control group and a sample of healthy subjects. Based on the previous work, the primary objective of this study was to propose an automated classification model of severity in ventricular contraction dyssynchrony. This model includes clinical data (LVEF, QRS, and P-R intervals) and the 3-MSF extracted from FADS of ERNA images representing the mechanical behavior of cardiac contraction. We believe that combining clinical data and ventricular mechanical descriptors may be helpful to select HF patients most likely to respond to CRT and will allow following a specific treatment.

## 2. Methods

### 2.1. ERNA Images Analysis and FADS

An ERNA study is a set of images representing the spatial distribution of a radionuclide inside the ventricular cavities at a specific time of the heart cycle and relates pixel intensity with ventricular volume.

**Figure 1:**
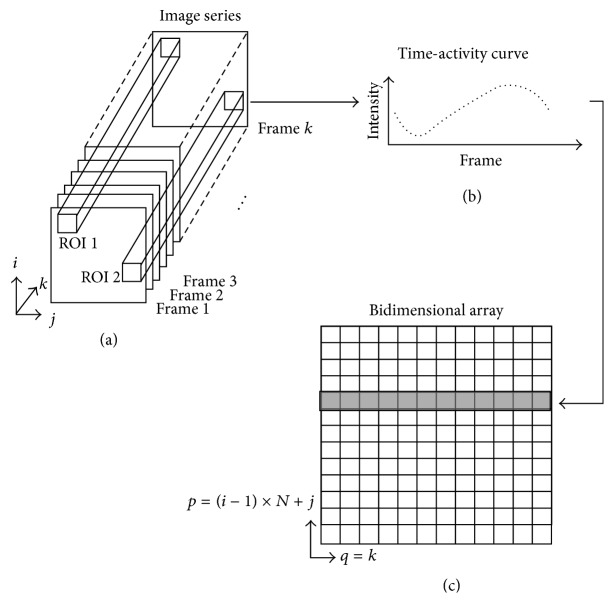
(a) depicts a ERNA study consisting of a* k*-images series, with frames having *i* × *j* pixels. (b) shows the time-activity curve extracted from a particular Region of Interest (ROI). (c) depicts a bidimensional array constructed from the image series. Figure adapted from [40].

Let *X*_TAC_(*p*, *q*) = *X*(*p*(*i*, *j*), *q*) be a bidimensional array (Figure 1(c)), a whole frame is stored in one column. For the *k*th frame of the acquired image series (*q* = *k*), the value of the (*i*, *j*)th pixel (Figure 1(a)) is located in the row *p* = (*i* − 1) × *N* + *j*. ERNA images were acquired with 16 frames of 64 × 64 pixels each one; thus *p* ∈ {0,1,…, 4095} and *q* ∈ {0,1,…, 15}. One row in *X*_TAC_(*p*, *q*) represents a time-activity curve (Figure 1(b)).

The FADS assumes that TAC curves from an ERNA study are a linear transformation of factors describing the dynamics of independent regions. FADS enhances pixel groups with a similar time evolution and additionally describes the geometry and spatial location of these pixel groups. Thereby, this model can be described by

(1) $$\begin{array}{c} X_{TAC}\left(\;p,q\;\right)=\Gamma C^{T}, \end{array}$$

where *X*_TAC_ is the bidimensional array of the series of *k* images from the ERNA study, **C** is the eigenvector matrix, extracted from covariance matrix of *X*_TAC_, which contains the FADS coefficients, and Γ is the principal components matrix (factors matrix from FADS).

### 2.2. Studied Population

A control group of 33 healthy volunteers was used in this study (21 males and 12 females) with mean age of 28 ± 5 years, LVEF of 59.66% ± 5.85%, and low probability for coronary artery disease [42]. After performing a thorough clinical evaluation, cardiac function was considered as normal, and this set of ERNA images were used as reference of normal contraction pattern.

A HF group of 42 subjects (32 males and 10 females) with mean age of 53.12 ± 15.05 years, LVEF < 35%, was studied, all of them clinically indicated for an ERNA study and followed at Instituto Nacional de Cardiología.  Table 1 summarizes the main clinical features of control and HF groups.

All participants were volunteers and gave informed consent to participate in the study according to the Helsinki declaration [44].

QRS and P-R intervals were taken from the clinical file of each participant, particularly from the closest electrocardiogram trace before the ERNA study. For the control group, electrocardiogram traces were not available so normality values reported in literature were assumed. All individuals gave their informed consent to participate in this study.

### 2.3. ERNA Images Acquisition

The same* General Electric Millennium MPR/MPS*® gamma camera was used for all ERNA images acquisition. The camera contains a single head with 64 photomultiplier tubes and it is equipped with a low-energy high-resolution parallel-hole collimator; the calibration of the energy peak was centered at 140 KeV and the detector uniformity was guaranteed at less than 5%. Images were digitized at a 64 × 64-pixel resolution and 1.33 as zoom factor.

Erythrocytes were tagged applying an in vivo/in vitro modified technique with 740 to 925 MBq of Tc-99m, using an UltraTag® RBC kit [45, 46]. Electrocardiogram trace was continuously monitored to synchronize images acquisition with the R wave. To eliminate ventricular extrasystoles during acquisition, a beat acceptance window was defined at ±20% of the average heart rate. Images were taken in an anterior left oblique projection to attain the best definition of left and right ventricles simultaneously. A total of 16 frames were obtained with a density of 300,000 counts per frame; however, in the analysis the last frame of each study was eliminated due to the low quality of the image as a result of the R-R interval variability during the acquisition, which leads to a low signal-noise ratio.

ERNA studies of control population were taken from a database acquired at 16 frames whereas patient studies were acquired at 32; to adjust dimensions between frames, a reduction was made for patient group in which the two consecutive frames were averaged (1 and 2, 3 and 4,…, 31 and 32) for having homogeneous sets at 16 images.

### 2.4. Labels for Study Population

Interventricular (LV-RV, between right and left ventricles) and intraventricular (iLV, inside left ventricle) dyssynchrony were visually evaluated by three nuclear cardiologists using the Fourier phase images [47] with prior knowledge of LVEF, QRS, and P-R interval of the control and HF groups. Dyssynchrony degree was coded as absent (*A*), mild (*M*), moderate (Md), and severe (*S*). Both LV-RV and iLV dyssynchrony labels were determined as the mode of the expert evaluations. In the cases where the mode was undetermined, the label was considered as the median of experts codes.

### 2.5. Classification Algorithm

In this study linear support vector machines (LSVM) of type C-SVM were implemented using the e1071 package in R based on LibSVM [48, 49].

**Figure 2:**
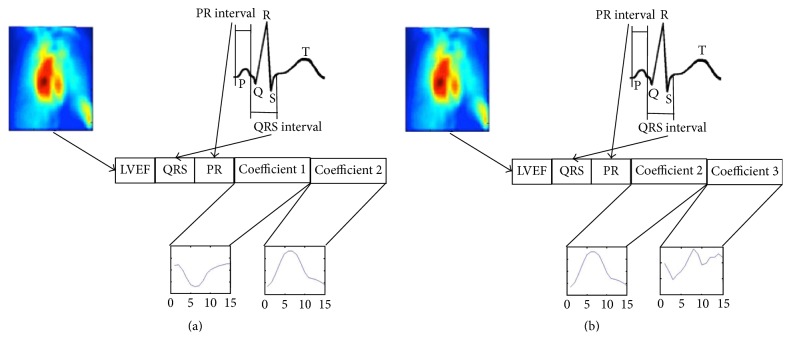
Features vectors configuration for SVM. (a) iLV dyssynchrony severity classification; (b) LV-RV dyssynchrony severity classification.

Feature vectors utilized in the SVM were built with information obtained from FADS plus LVEF, QRS, and P-R intervals from each participant. There were defined two classification systems, one for iLV dyssynchrony and other for LV-RV dyssynchrony. The configuration of the characteristics vectors in each case is shown on Figure 2.

The first three values of the vector correspond to LVEF (normalized percentage calculated from ERNA [25]), QRS, and P-R intervals, respectively (measured in milliseconds). According to the findings in [30] the following data are the values from curves describing first and second coefficients of FADS for iLV dyssynchrony case and second and third coefficients of FADS for LV-RV dyssynchrony. More information from this analysis was not incorporated because it has been demonstrated that the three most significant factors contain more than 95% of information of the ERNA images [41].

Cardiologist suggested combining the classes of dyssynchrony severity into the following way: one stage to distinguish healthy participants (absent dyssynchrony) versus nonhealthy participants (present dyssynchrony) and a second stage to distinguish in between nonhealthy participants, those who have a mild dyssynchrony versus those with a moderate or severe dyssynchrony (nonmild). The distinction between the latter two is not necessary.

**Figure 3:**
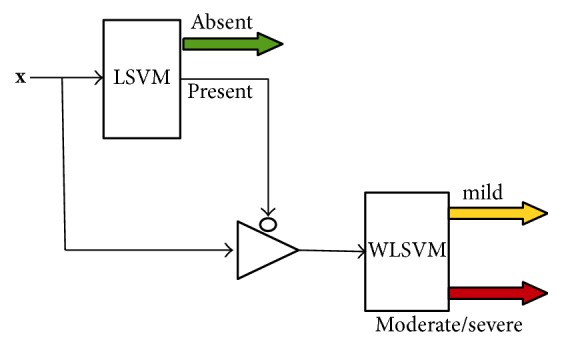
Classification scheme for each type of dyssynchrony. **x**: feature vector (including LVEF, QRS, P-R, and FADS information). LVSM: linear support vector machine. WLSVM: weighted linear support vector machine.

Once the features were defined, the classification scheme was selected; two binary linear SVM (LSVM) coupled in cascade were implemented: the former to classify absent versus present dyssynchrony patterns and the latter (for cases with dyssynchrony) to distinguish mild or moderate-severe dyssynchrony. The proposed scheme is shown in Figure 3.

On iLV dyssynchrony, for the first LSVM, we had balanced classes with absent/present dyssynchrony subjects (45%/55%); however for the second SVM a weighted linear SVM (WLSVM) was used due to an unbalance in mild/moderate to severe cases (15%/85%). On LV-RV dyssynchrony, for the first LSVM, we also have balanced classes of absent/present dyssynchrony subjects (44%/56%) and for the second WLSVM we also have unbalanced mild/nonmild classes (26%/74%).

Data were divided into training and testing groups on a 70%/30% proportion. Cross-validation was used in order to evaluate the LSVM performances; it consisted of dividing the training data into subgroups and alternating them as training and testing sets to finally compute the average of classification results [50].

The last step in SVM's implementation is to test the classifier's performance with a set of cases nonseen into the training stage. Finally, the confusion matrix was built comparing the results from automatic model with those achieved by the physicians, rows of matrix show true labels (expert's classification), and columns show the model's prediction. The global classification results are shown in an extended table at the end of results section.

## 3. Results

### 3.1. Factor Analysis of Dynamic Structures

**Figure 4:**
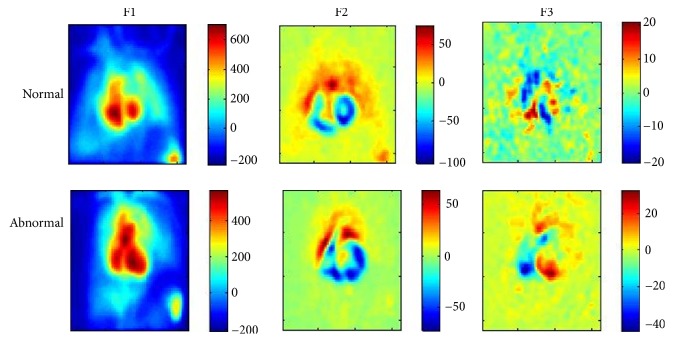
Distribution of the three most significant factors (F1, F2, and F3) computed from a normal and an abnormal ERNA set of images.

Figure 4 shows the three most significant factors computed from FADS for a healthy participant and one from the HF group. For the first one, the maximum values in the first factor (F1) are located within the ventricular area, whereas in the second (F2) the maximum values are found in the atrial area and the minimum ones in the ventricular region. In the third factor (F3) the maximum values are distributed with a nonanatomical region associated. On the second, images of abnormal contraction pattern show a similar behavior for factors F1 and F2 with maximum values in ventricular region for the first one, whereas the second one shows maximum and minimum values in atrial and ventricular areas, respectively. Nevertheless, the third factor (F3) shows a distribution of both maximum and minimum values within the ventricular cavities region.

A contribution of factors associated with each coefficient showed that more than 99% of information (see Table 2) is contained in the first three most significant factors.

### 3.2. Patient Classification by Nuclear Cardiologists

There was a significant agreement evaluated with Kendall's coefficient of concordance between three nuclear cardiologists. They only knew the clinical data (LVEF, QRS, and P-R intervals) and the Fourier phase image to tag each case of LV-RV dyssynchrony (0.8, *p* < 0.001) and iLV dyssynchrony (0.59, *p* < 0.001) into absent, mild, moderate, or severe classes.

Labels of cardiac dyssynchrony degree for the studied population computed as the mode of 3 nuclear cardiologists evaluations (see Section 2.4) are shown in Table 3.

### 3.3. Classification Model

#### 3.3.1. Training and Testing Sets

SVM performance was evaluated by a 10-fold cross-validation procedure from the training set.  Table 4 shows the mean accuracy and standard deviation for each stage on classification model described in Section 2.5.

#### 3.3.2. Optimal Classification Model

The support vector machines were built from a unique training set and results shown in Table 4 are an amount of trials and average values from many SVM's performances that allow us to observe a trend on how this kind of algorithm works solving this particular problem. The values of parameters gamma (*γ*) and cost function (*C*) were chosen as those that achieved the best performance for each classifier; in the first SVM (iLV) these values were 0.15 and 65 whereas for the second (LV-RV) they were 0.05 and 10, respectively.

For LV-RV dyssynchrony the testing set was formed with 10, 4, and 10 participants from absent, mild, and moderate-severe labels, respectively, whereas for iLV dyssynchrony the testing set was formed with 11, 2, and 11 participants from absent, mild, and moderate-severe labels, respectively.  Table 5 shows the results of the classification of testing set.

The categories best classified were absent for both LV-RV and iLV with the 100% and 90% of true positive rates, respectively, and moderate-severe for iLV dyssynchrony with a 90.91% hit rate.

The SVM proposed that merge FADS and clinical data have hit rates of 90%, 50%, and 80% to distinguish between absent, mild, or moderate-severe interventricular dyssynchrony, respectively. For intraventricular dyssynchrony, hit rates of 100%, 50%, and 90% differentiated between absent, mild, or moderate-severe, respectively, in a testing set. In all cases, the mean accuracy reached was at least of the 75% to distinguish mild and nonmild dyssynchrony and 96% to distinguish absent and present dyssynchrony.

## 4. Discussion

The findings regarding spatial distribution of the 3-MSF confirm the results of Jiménez-Ángeles et al. [40], where the same maximum and minimum values distribution over the mentioned factors was described. For that study, the population was one group of control participants and two groups of patients with a clinically established diagnosis. One included patients with dilated cardiomyopathy and the other patients with complete left bundle branch block. For results shown in the present work, the clinical group was not classified by a particular cardiomyopathy; the condition that all patients share is heart failure. Additionally, results from Table 2 justify using only the 3-MSF for the construction of the automated classification model.

The SVM included information obtained from FADS as features, whereas the labels were assigned using phase image analysis. This may introduce variability within the model for using different sources of information; however, contraction pattern evaluation in clinical practice uses it. Although FADS have been shown to be superior to Fourier phase image [38, 40], it is still a method under evaluation.

In general terms it can be noted that SVM algorithm offers good results getting a classification hit rate close to 80% for LV-RV dyssynchrony, having 1 false positive on the first stage (absent versus present) and four mistakes for second stage (mild versus moderate-severe). The hit rate for iLV was 100% on the first stage (absent versus present) and had 2 mistakes for the second stage (mild versus moderate-severe). It is possible to raise the hit rate for the mild class if the number of patients in this group is increased. Thus, it will be defined better, the latter being relevant in order to dictate the corresponding follow-up and clinical treatment. As HF patients were collected from Mexican National Center of reference, normal or mild abnormalities are the least frequent cases and they are not necessarily indicated for a nuclear medicine study. Hence, this study opens an opportunity for multicenter trials to confirm our preliminary results.

The classification model herein presented was built on the agreement of three nuclear cardiologists who classified in mild and moderate-severe the left ventricular and interventricular dyssynchrony in a double-blind way using only the phase image and clinical data from each patient. Thus, it is built on clinical agreement.

### 4.1. Study Limitations

In spite of the relatively low simple size in this study, it presents an automated method for classifying the severity of cardiac contraction dyssynchrony with a reliable performance that warrants further prospective studies. Also, age difference between the control group and patient population is too discrepant and that may influence the results. Inclusion criteria for the control volunteers were chosen considering a low pretest likelihood of coronary artery disease, no known history of cardiac disease, and normal sinus rhythm in the electrocardiogram signal. However, all of these features increase their prevalence and incidence with age [42, 51]. In order to have a representative sample of healthy participants that meet all the inclusion criteria with clinical indication for ERNA, image acquisition was performed at rest. Port et al. reported the effects of age on the LVEF, end-diastolic volume, and regional wall motion using ERNA at rest and exercise in 77 healthy volunteers between 20 to 95 years of age concluding that age did not appear to influence any of these measurements at rest [52].

Additionally, recent studies have indicated that regional scar tissue and global scar burden may also be related to nonresponse to CRT [52]. Data on scar tissue and/or segmental wall motion abnormalities was not systematically available and was not incorporated in the classification algorithm.

## 5. Conclusion

Using a reproducible, minimally invasive, and low-cost nuclear medicine method such as ERNA plus relevant clinical information extracted from the ECG, we have proposed an automated classification method for severity of cardiac dyssynchrony with reliable performance. Its main features include an improved analysis of the severity in left-intraventricular and interventricular dyssynchrony, differentiation of mild and nonmild HF patients, and the inclusion of nuclear cardiologists expertise to define the categories. Thus, this model seems promising in the clinical follow-up of CRT or in a particular treatment.

## Figures and Tables

**Table 1:** Clinical features from studied population. (1) Patients without ischemic cardiomyopathy and QRS > 120 ms. (2) Patients without ischemic cardiomyopathy and QRS < 120 ms. (3) Patients with ischemic cardiomyopathy and QRS > 120 ms. (4) Patients with ischemic cardiomyopathy and QRS < 120 ms. ^*∗*^Normality values reported in literature [43].

Group	LVEF%	QRS [ms]	P-R [ms]	Number of subjects
Control	59.7 ± 5.8	80^*∗*^	140^*∗*^	33
(1) Non-Isch. QRS > 120 ms	22.1 ± 7.4	150.9 ± 30.1	178.18 ± 34.9	11
(2) Non-Isch. QRS < 120 ms	23.3 ± 8.2	88.6 ± 8.8	191 ± 23.8	10
(3) Isch. QRS > 120 ms	22.6 ± 10.6	139.0 ± 23.8	191 ± 23.8	10
(4) Isch. QRS < 120 ms	32.9 ± 9.3	95.0 ± 12.4	176.36 ± 43.9	11

LVEF: left ventricle ejection fraction.

**Table 2:** Contribution of first three most significant factors for control and HF groups.

Group	Contribution (%)
Control	99.77 ± 0.08
HF	99.78 ± 0.11

HF: heart failure.

**Table 3:** Labels of cardiac dyssynchrony degree for all subjects computed as the mode of 3 nuclear cardiologists visual evaluations.

Dyssynchrony		LV-RV	iLV
		(Number of subjects)	(Number of subjects)
Absent		33	34
Present	Mild	11	6
	Moderate/severe	31	35

LV-RV: interventricular dyssynchrony and iLV: intraventricular dyssynchrony.

**Table 4:** Mean accuracy of classifiers.

Classes	LV-RV	iLV
	Dyssynchrony	Dyssynchrony
Absent/present	96.10% ± 9.33%	99.96% ± 0.82%
(LSVM)		
Mild/moderate-severe	78.17% ± 23.26%	75.55% ± 23.98%
(WLSVM)		

LSVM: linear support vector machines and WLSVM: weighted linear support vector machine.

**Table 5:** Classification results for testing set showed as percentage and number of subjects in parenthesis.

Dyssynchrony	Absent	Mild	Moderate-severe	Total
LV-RV	90%	50%	80%	79.17%
	(9/10)	(2/4)	(8/10)	(19/24)
iLV	100%	50%	90.91%	91.67%
	(11/11)	(1/2)	(10/11)	(22/24)
